# Supplementary material for: Predicting visual function by interpreting a neuronal wiring diagram
Source: Nature. 2024 Oct 2;634(8032):113–23. doi: 10.1038/s41586-024-07953-5 (PMC11446822; doi:10.1038/s41586-024-07953-5)
Supplement: Supplementary file 6 — CRF and ERF predictions for individual TmY4 and TmY9 cells. Analogous to Supplementary Data 3, but for TmY target types. Shown are the top four monosynaptic pathways, the strongest pathway passing through each of the top ten intermediary types (ranking from Extended Data Fig. 7), and the trisynaptic pathway Tm1–TmY–Dm3–TmY (see the section entitled Prediction of spatial normalization). [file 41586_2024_7953_MOESM6_ESM.zip › DataS4/TmY9q/720575940623527143.pdf]

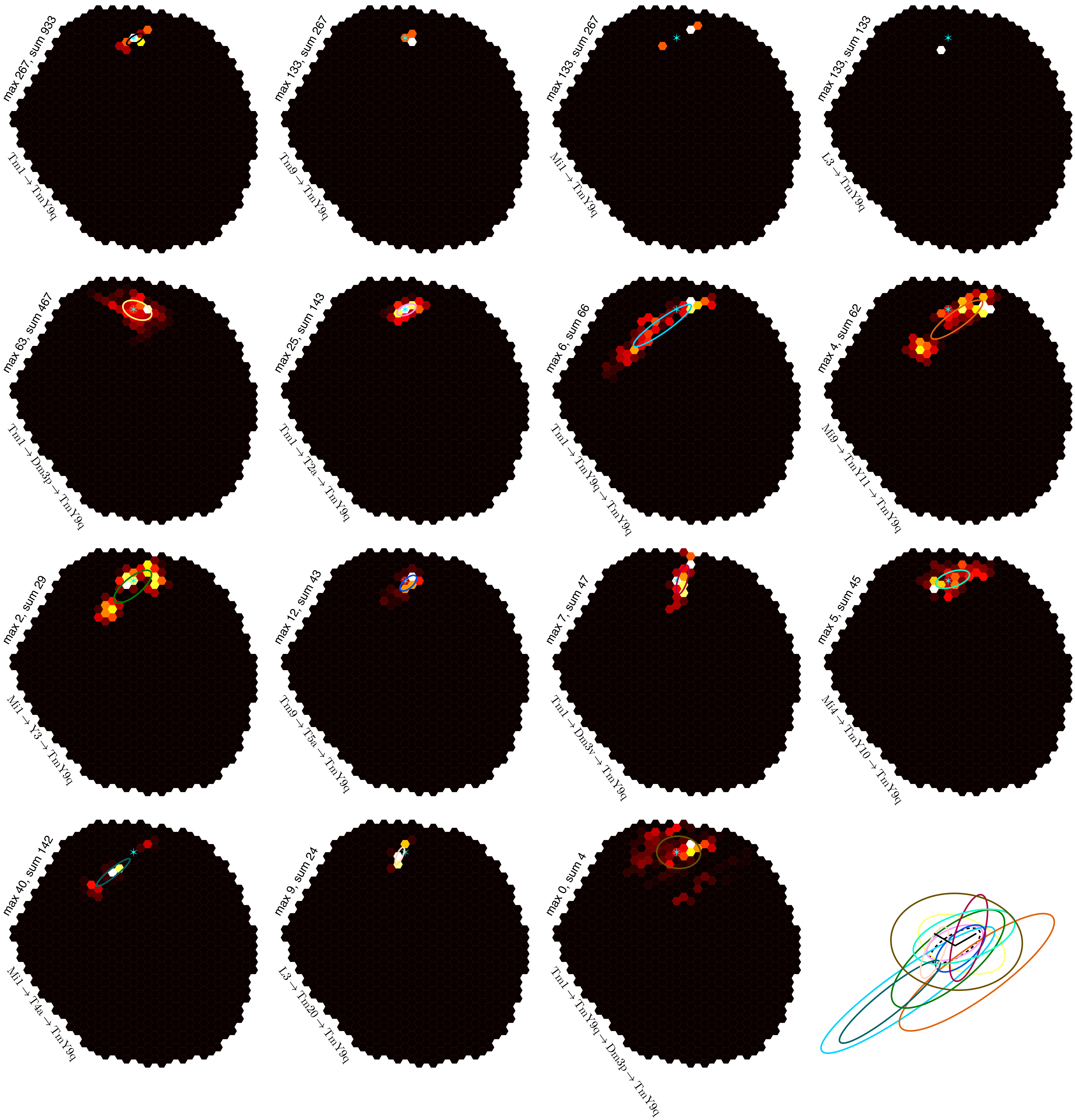
max 267, sum 933
Tm1 → TmY9q
max 133, sum 267
Tm9 → TmY9q
max 133, sum 267
Mi1 → TmY9q
max 133, sum 133
L3 → TmY9q
max 63, sum 467
Tm1 → Dm3p → TmY9q
max 25, sum 143
Tm1 → T2a → TmY9q
max 6, sum 66
Tm1 → TmY9q → TmY9q
max 4, sum 62
Mi9 → TmY11 → TmY9q
max 2, sum 29
Mi1 → Y3 → TmY9q
max 12, sum 43
Tm9 → T5a → TmY9q
max 7, sum 47
Tm1 → Dm3v → TmY9q
max 5, sum 45
Mi4 → TmY10 → TmY9q
max 40, sum 142
Mi1 → T4a → TmY9q
max 9, sum 24
L3 → Tm20 → TmY9q
max 0, sum 4
Tm1 → TmY9q → Dm3p → TmY9q
